# Delayed Exposures and Pre‐Exposure Periods in Self‐Controlled Case Series Studies

**DOI:** 10.1002/sim.70566

**Published:** 2026-04-27

**Authors:** Heather Whitaker, Yonas Ghebremichael Weldeselassie, Paddy Farrington

**Affiliations:** ^1^ Modelling Division UK Health Security Agency London UK; ^2^ School of Mathematics and Statistics The Open University Milton Keynes UK; ^3^ Warwick Medical School University of Warwick Coventry UK

**Keywords:** adverse event, bias, exposure delay, pre‐exposure period, self‐controlled case series, vaccine

## Abstract

A key assumption of the self‐controlled case series (SCCS) method is that exposures should not depend on the event of interest. However, treatments such as vaccines may be deferred after an adverse health event. One suggestion to handle such delayed exposures is to include a pre‐exposure window in the SCCS model. We study the impact of such adjustments and of exposure deferment on the SCCS relative incidence estimates. We obtain explicit results in a simplified setting, and investigate more realistic scenarios by simulation. We develop some practical recommendations for sensitivity analyses: when the delayed exposures remain within the observation period, no adjustment is needed. When exposures are delayed beyond the end of the observation period, an adjustment may be required. In some circumstances the SCCS model for event‐dependent exposures should be used rather than the standard SCCS model. These options are illustrated with three practical examples.

**Abbreviations**
GBS: Guillain Barré syndrome
MMR: measles mumps rubella
OPV: oral polio vaccine
SCCS: self‐controlled case series

## 1 Introduction

The purpose of the self‐controlled case series (SCCS) method is to estimate the association between an exposure and a disease outcome, and is most often used in pharmacoepidemiology with transient drug exposures. The method uses a conditional likelihood involving only cases and within‐patient comparisons across successive time periods, and is therefore unaffected by time‐invariant multiplicative confounders or frailties [1, 2].

However, exposures must not depend on the outcome. This condition is violated if treatment is delayed after an event. For example, if the exposure is measles vaccination and the event of interest is febrile convulsion, vaccination may be delayed in children who have experienced a convulsion shortly before they were due to receive the vaccine. Some vaccinations may then occur some time later than planned; occasionally, vaccination appointments may not be rescheduled, and the child may remain unvaccinated.

The purpose of the present paper is to study in greater detail the impact of delayed exposures, and to determine an optimal strategy to handle them. We consider exposure delays that may be initiated within a relatively short time period after an event: we refer to this short time period as the deferment interval. To handle brief deferment intervals in SCCS studies, one proposed strategy is to include a pre‐exposure ‘risk’ period prior to the exposure [2]. The rationale is that such a pre‐exposure period would correct the dearth of events occurring shortly before the exposure, which might bias the relative incidence upwards. In this paper, we study this type of adjustment in greater detail than hitherto. We show that, in many circumstances, such an adjustment is not necessary, and may in fact introduce bias. However, in other circumstances, bias does arise from delays, and including an appropriate pre‐exposure period will correct it.

In order to situate the present development, we begin in the next section with a brief review of work done so far on event‐dependent exposures in the context of SCCS studies. In subsequent sections, we motivate our present investigation by describing two special scenarios, with simple numerical illustrations. We then develop a model for event‐related exposure delays in a simple setting, which provides a basis for possible adjustments. These are studied by simulation in realistic settings. We then set out some practical recommendations, which we illustrate with three contrasting examples.

## 2 SCCS Models and Event‐Dependence

A formal derivation of the SCCS model has been given elsewhere and will not be repeated here [1, 3]. Suffice it to say that the model is derived from a Poisson cohort model by conditioning on the number of outcomes occurring for each individual within a pre‐specified observation period. However, for the association parameter of interest (between exposure and event) to retain its cohort interpretation in the conditional model, exposures and observation periods must not depend on outcomes. In particular, the exposure must be an external, or exogenous, variable, and observation periods must be independent of outcomes (see, in particular, the contribution to the Discussion in Reference [3] by Neils Keiding) [3].

Much previous work has focused on weakening or circumventing these restrictive requirements in situations that have arisen in pharmacoepidemiology. For example, if the outcome of interest is an event that precludes the exposure occurring at any subsequent time (as is the case with intussusception and vaccination against Rotavirus, intussusception being a contraindication to vaccination), a modified SCCS method eschewing the likelihood framework and based instead on estimating equations may be used [4, 5]. If the outcome of interest is death or is an event which is associated with increased mortality (both of which may lead to event‐dependent observation periods), further modified SCCS models may be used in certain circumstances [6, 7].

In the present paper, we consider a less extreme scenario, in which the dependence of the exposure arises only in a brief time interval after the outcome event, and observation periods are not event‐dependent. An early investigation of this setting involved dependent outcome and exposure processes [3]. It was shown that, in certain circumstances, a simple correction term may be incorporated into the standard SCCS model by including a brief pre‐exposure period (see Proposition 2 of Reference [3]). However, this setting does not align with vaccinations, which are typically undertaken according to recommended schedules, and usually involve advance booking of vaccination appointments or prior planning of vaccination campaigns.

In the present paper, we focus on the following more realistic scenario. Exposure times (such as vaccinations) are determined in advance (e.g., by booking appointments), and if an exposure happens to have been planned within a set period after an event, this exposure may be delayed or canceled. Since about 2001, it has been suggested that this situation could be handled by including a short pre‐exposure period within the standard SCCS model, for example as part of a sensitivity analysis [8, 9]. However, simulations have shown that the inclusion of pre‐exposure periods can introduce or overcorrect the bias when exposures are briefly delayed rather than canceled [10]. Our aim is therefore to seek to clarify the issues involved and to investigate such adjustments more systematically.

## 3 Motivating Scenarios

In this section, we describe some simple scenarios that show that brief exposure deferment intervals may or may not introduce bias in the SCCS method; and that including a pre‐exposure period may or may not help.

**FIGURE 1:**
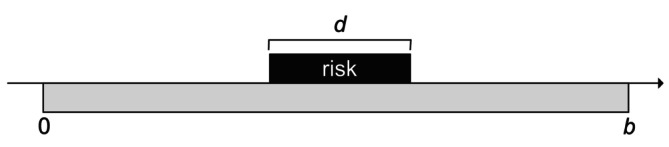
Basic set up.

Suppose first that there are N cases, each with the same observation period of duration b, each with a single event, and each with a post‐exposure risk period of duration d. The parameter of interest is the relative incidence that is the ratio of the incidence in the risk period to that in the control period (the control period being all time within the observation period not included within the risk period). This scenario is represented in Figure 1.

In the absence of age or temporal effects, the SCCS log likelihood contribution of a single case i is

(1) $$l_{i}\left(\beta \right)=R_{i}\beta -\log\left\{e^{\beta }d+\left(b-d\right)\right\},$$

where β is the log relative incidence and $R_{i}$ is the indicator random variable taking the value 1 if the event occurs in the risk period and 0 if the event occurs in the control period. If there are $N=N_{0}+N_{1}$ cases, with $N_{0}$ events in the control period and $N_{1}$ events in the risk period, the maximum likelihood estimator of the relative incidence is

(2) $$e^{\hat{\beta }}=\frac{N_{1}}{N_{0}}\times \frac{b-d}{d}.$$

For example, suppose b=100 days, d=10 days, β=0 (corresponding to no association). As the number of cases grows large, $N_{1}/N_{0}$ tends to d/(b−d)=10/90 and the estimated relative incidence tends to 1, as expected.

**FIGURE 2:**
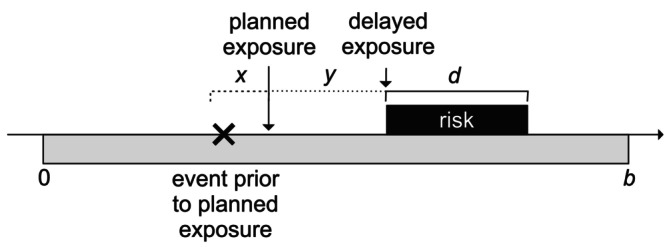
Delay of exposure by y days.

Now suppose that if an event occurs within x days prior to a planned exposure, that exposure is delayed by a period y≥x days, but that the risk period remains completely within the observation period. A planned exposure might, for example, be a vaccination appointment. The situation is represented in Figure 2.

In our numerical example, suppose from now on that exposures are planned at times between days 10 and 80 of the observation period, and y=x=10 days. Thus, with short delays, actual exposures will occur between days 10 and 90 of the observation period. Each case will thus still experience a risk period of duration 10 days and a control period of duration 90 days; the numbers of cases in the risk and control periods are unaffected. In consequence, Equations (1) and (2) still apply, and the estimated relative incidence is unaffected.

**FIGURE 3:**
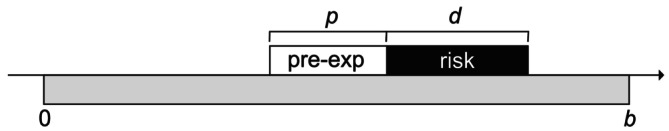
Including a pre‐exposure period of duration p.

Now suppose that, in the presence of the delays just described, a pre‐exposure risk period of duration p≤x days is introduced. The situation is now represented in Figure 3.

Owing to the delays, there are zero events in the x=10 days preceding any risk period, and hence no events in the pre‐exposure periods. However, the control period is now of duration b−d−p days, since we need to subtract the p days in the pre‐exposure period. The estimated relative incidence is

(3) $$e^{\hat{\beta }}=\frac{N_{1}}{N_{0}}\times \frac{b-d-p}{d}.$$

In our numerical illustration, the counts $N_{1}$ and $N_{0}$ remain unchanged, and $N_{1}/N_{0}$ still tends to 1/9. When p=10 days, (b−d−p)/d=0.8/0.1. The estimated relative incidence is therefore 8/9≃0.889: using the pre‐exposure period has introduced a downward bias.

**FIGURE 4:**
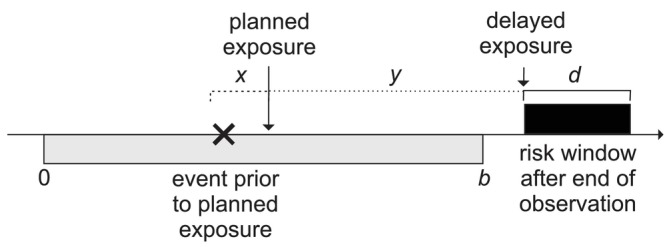
Delay beyond the end of observation.

We now consider a second scenario in which events occurring within x days before the planned exposure result in postponment of the exposure for y days, but now y is long, always removing the rescheduled exposure beyond the end of the observation period. This is represented in Figure 4.

Cases in whom the exposure is delayed are no longer exposed within the observation period. Within the SCCS model, these cases no longer contribute to the likelihood for β: this can be seen by setting $R_{i}=d=0$ in Equation (1). The remaining cases contribute as before. However, the number of events in the control period for these remaining cases is now $N_{0}^{*}<N_{0}$, and the relative incidence is estimated as

(4) $$e^{\hat{\beta }}=\frac{N_{1}}{N_{0}^{*}}\times \frac{b-d}{d}.$$

In our numerical example, the number of cases with events occurring within x=10 days of the planned exposure is $\left(10/90\right)\times N_{0}$ and so $N_{1}/N_{0}^{*}$ now tends to 1/(9×(1−1/9))=1/8. Thus the estimated relative incidence tends to (1/8)×9=1.125. So the relative incidence is biased upwards.

Now suppose that we reinstate the pre‐exposure period represented in Figure 3, with p≤x. The relative incidence now becomes

(5) $$e^{\hat{\beta }}=\frac{N_{1}}{N_{0}^{*}}\times \frac{b-d-p}{d}.$$

In our numerical example with p=10 days, $N_{1}/N_{0}^{*}$ remains as before, but the control period is shortened. The relative incidence is now (1/8)×8=1: introducing the pre‐exposure period has removed the bias.

We also investigated scenarios in which delayed exposures result in risk periods that are only partly contained within the observation period. However this leads to a more complicated expression that provides little further insight (see Supporting Information). The assumption that risk periods are completely contained within the observation period (or completely excluded) is not an unreasonable one for childhood or seasonal vaccination, which is typically recommended to follow a specified schedule. Observation periods are then chosen to contain these scheduled times. Note however that the standard SCCS model readily accommodates varying risk periods and pre‐exposure periods, as well as age effects. The simulations in Section 5 will aim to study exposure delays in this less constrained setting.

## 4 A Model for Delayed Exposures

In the present section we develop a model for delayed exposures that generalizes the scenarios previously illustrated, while retaining some of their simplicity so it remains tractable. Specifically, we assume that all cases have the same observation period of duration b, a single planned risk period of duration d, and a single event; the risk period will typically be much shorter than the observation period. To ensure tractability, there are no age or period effects. We also assume that actual risk periods are completely contained within the observation period or completely excluded. Similarly, all pre‐exposure periods are completely included within the observation period.

If a planned exposure falls within a period x after the event (this period is the deferment interval), one of three things can happen. First, with probability π, the exposure is delayed, and with probability 1−π, it occurs as planned. Given that the exposure is delayed, with probability ϕ, the exposure is delayed beyond the end of the observation period (or canceled entirely), while with probability 1−ϕ, it is delayed by a period y≥x, such that the risk period remains entirely within the observation period. Delays with these characteristics will be referred to as ‘long’ and ‘short’, respectively. Thus, the three possibilities are: no delay (which occurs with probability 1−π), long delay (with probability πϕ), and short delay (with probability π(1−ϕ)). In this model, event‐dependence arises from the fact that whether or not the exposure can be delayed depends on the timing of the event. However, whether the delay is brief or long (given that a delay occurs) is not event‐dependent.

A SCCS model is then fitted with exposure risk period of duration d, and pre‐exposure risk period of duration p. We assume that p≤x≤y. The relative incidence associated with the risk period is exp(β). The relative incidence associated with the pre‐exposure period is formally represented by exp(γ).

The baseline incidence rate is denoted λ, though this factors out of the SCCS likelihood; λ is small, consistent with each case including a single event; if there is more than one event per case, we restrict attention to the first event. Suppose there are N cases. These N cases may then be partitioned as follows into six groups, according to when the event occurs.

1. $N_{1}$ events within the risk period. The SCCS likelihood contribution of these events under the fitted model is:
$$L_{1}={\left(\frac{\exp\left(\beta \right)d}{\exp\left(\beta \right)d+\exp\left(\gamma \right)p+b-d-p}\right)}^{N_{1}}$$
  and the expected value of $N_{1}$ is λexp(β)d.
2. $N_{2}$ events within the deferment interval x, with exposure delayed by a short period y. Their likelihood contribution is:
$$L_{2}={\left(\frac{x}{\exp\left(\beta \right)d+\exp\left(\gamma \right)p+b-d-p}\right)}^{N_{2}}$$
  and the expected value of $N_{2}$ is λxπ(1−ϕ).
3. $N_{3}$ events within the deferment interval x, with exposure delayed beyond the end of observation. Their likelihood contribution is:
$$L_{3}={\left(\frac{x}{b}\right)}^{N_{3}}.$$
  The expected value of $N_{3}$ is λxπϕ; these events are not directly informative about the parameters of interest.
4. $N_{4}$ events within the deferment interval of length x, with exposure not delayed, but occurring within the period of length x−p not included in the pre‐exposure interval. Their likelihood contribution is:
$$L_{4}={\left(\frac{x-p}{\exp\left(\beta \right)d+\exp\left(\gamma \right)p+b-d-p}\right)}^{N_{4}}$$
  and the expected value of $N_{4}$ is λ(x−p)(1−π).
5. $N_{5}$ events within the deferment interval of length x, with exposure not delayed, and occurring within the pre‐exposure interval of length p. Their likelihood contribution is:
$$L_{5}={\left(\frac{\exp\left(\gamma \right)p}{\exp\left(\beta \right)d+\exp\left(\gamma \right)p+b-d-p}\right)}^{N_{5}}$$
  and the expected value of $N_{5}$ is λp(1−π).
6. $N_{6}$ events outside both the risk period and the deferment interval. Their likelihood contribution is:
$$L_{6}={\left(\frac{b-d-x}{\exp\left(\beta \right)d+\exp\left(\gamma \right)p+b-d-p}\right)}^{N_{6}}$$
  and the expected value of $N_{6}$ is λ(b−d−x).

Note that delayed exposures within cases does not result in dependence between cases. The log‐likelihood of the N cases for the fitted model is then

$$l\left(\beta ,\gamma \right)=\sum_{i=1}^{6}N_{i}\log\left(L_{i}\right).$$

Maximizing the log‐likelihood with respect to the parameters β and γ yields the following estimators:

(6) $$\begin{array}{rr} \exp\left(\hat{\beta }\right) & =\frac{N_{1}/d}{\left(N-N_{1}-N_{3}\right)/\left(\exp\left(\hat{\gamma }\right)p+b-d-p\right)},\\ \exp\left(\hat{\gamma }\right) & =\frac{N_{5}/p}{\left(N-N_{3}-N_{5}\right)/\left(\exp\left(\hat{\beta }\right)d+b-d-p\right)}. \end{array}$$

We are interested in the asymptotic limits of these estimators as N→∞, which are obtained by substituting the expected values of the event counts. Denoting these limits by tildes, we obtain:

(7) $$\begin{array}{rr} \exp\left(\tilde{\beta }\right) & =\exp\left(\beta \right)\left(1+\frac{x\pi \phi -p\left(1-\exp\left(\tilde{\gamma }\right)\right)}{b-d-x\pi \phi }\right),\\ \exp\left(\tilde{\gamma }\right) & =\left(1-\pi \right)\left(\frac{\exp\left(\tilde{\beta }\right)d+b-d-p}{\exp\left(\tilde{\beta }\right)d+b-d-p+\pi \left(p-\mathrm{x\phi}\right)}\right). \end{array}$$

These equations may be used to study the asymptotic bias. Suppose first that all event‐dependent delays in exposure are short (ϕ=0). Then if no pre‐exposure period is included (p=0), there is no bias. If a pre‐exposure period is included, $\exp\left(\tilde{\beta }\right)<\exp\left(\beta \right)$, so the association parameter is biased downwards towards zero. But suppose now that some event‐dependent delays are long, and move exposures beyond the end of observation (ϕ>0). Then if no pre‐exposure period is included (p=0), $\exp\left(\tilde{\beta }\right)>\exp\left(\beta \right)$, so the association parameter is biased upwards. When all delays are long (ϕ=1), including a pre‐exposure period p=x removes this bias. Thus, these results confirm those of the previous section.

More generally, when there is a mix of delays, the optimal value of p is that for which $\exp\left(\tilde{\beta }\right)=\exp\left(\beta \right)$. This occurs when

(8) p=xϕ.

since in this case, we also have $\exp\left(\tilde{\gamma }\right)=1-\pi$.

Finally, note that, whatever the values of π and ϕ, and whether or not a pre‐exposure period is used, the relative asymptotic bias $\left(e^{\tilde{\beta }}-e^{\beta }\right)/e^{\beta }$ is bounded by x/(b−d−x). It may thus be reduced by increasing b, the duration of the observation period. Therefore, the bias can be controlled by design: longer observation periods are recommended.

## 5 Simulations

We studied the impact of exposure delays and pre‐exposure periods by simulation. We included age effects and risk periods only partly contained within the observation period; these were precluded in the analytical development of the previous section, in order to keep the calculations simple.

Each run involves a sample size N=500 cases with a single planned exposure. All cases have observation periods 1 to 600 days, and a single event. The planned exposure times were generated using the uniform distribution on V={1,…,600}. The event times were generated using the binomial distribution on V, with probabilities determined by the post‐exposure risk period d, the relative incidence exp(β), and the age effect. The risk periods investigated were 15 days (short risk period), 50 days (long risk period), and 100 days (very long risk period). The relative incidence was 0.5, 1, or 2. Three age effects were investigated: none, doubling, and halving (over the whole observation period), in 5 steps of 120 days (1,1.25,1.5,1.75,2 and vice‐versa). If the planned exposure occurred within a deferment interval of x days after the event, the actual exposure time was defined as follows. With probability π it was delayed (and with probability 1−π it was set at its planned value). If it was delayed, then with probability ϕ it was delayed beyond the end of observation, and with probability 1−ϕ it was set at its planned value plus y=x days. The values x investigated were 15 days (short deferment interval), 50 days (long deferment interval), and 100 days (very long deferment interval). The values of π and ϕ were 0.2 and 0.8 (4 combinations of these 2 values).

Three SCCS models were fitted to each simulated set of 500 cases, using the R package SCCS [11]. All three included correctly specified risk period and age intervals. The first model included no pre‐exposure period. The second included a pre‐exposure period of optimal duration xϕ. The third included a pre‐exposure period of duration x. For each set of parameters, 1000 independent samples were generated and the medians of the parameters estimates corresponding to the risk period were obtained. Note that when there are zero events in the risk or control period, the estimate of β is infinite; accordingly we used the median of the simulated samples rather than the mean in all calculations.

The results, for scenarios without age effects, are in Tables 1, 2, 3. Also included are the Monte Carlo standard errors, which were the same to two significant figures for each value of β within each table. Inclusion of age effects did not alter the picture in any important way (the corresponding tables with age effects are in the Supporting Information). For ease of interpretation, median biases of absolute value at least 0.05 have been highlighted in bold type; these correspond to biases in the relative incidence of more than about 5%.

**TABLE 1:** Median bias of $\hat{\beta }$ with risk period d=15, for contrasting values of the relative incidence exp(β), deferment interval duration x, and delay probabilities π and ϕ.

	exp(β)=0.5 ^a^			exp(β)=1 ^b^			exp(β)=2 ^c^		
	Model 1	Model 2	Model 3	Model 1	Model 2	Model 3	Model 1	Model 2	Model 3
x=15									
π=0.2,ϕ=0.2	−0.039	−0.040	−0.043	−0.028	−0.028	−0.034	−0.001	−0.002	−0.006
π=0.2,ϕ=0.8	−0.036	−0.039	−0.040	−0.025	−0.029	−0.031	0.002	−0.001	−0.003
π=0.8,ϕ=0.2	−0.036	−0.040	**−0.056**	−0.025	−0.029	−0.046	0.002	−0.001	−0.019
π=0.8,ϕ=0.8	−0.023	−0.040	−0.044	−0.015	−0.029	−0.034	0.015	−0.001	−0.006
x=50									
π=0.2,ϕ=0.2	−0.036	−0.038	**−0.051**	−0.025	−0.029	−0.045	0.001	−0.002	−0.019
π=0.2,ϕ=0.8	−0.026	−0.037	−0.041	−0.016	−0.030	−0.036	0.010	−0.003	−0.009
π=0.8,ϕ=0.2	−0.021	−0.034	**−0.090**	−0.011	−0.025	**−0.081**	0.015	0.002	**−0.056**
π=0.8,ϕ=0.8	0.017	−0.038	**−0.051**	0.022	−0.031	−0.044	**0.051**	−0.004	−0.017
x=100									
π=0.2,ϕ=0.2	−0.028	−0.034	**−0.061**	−0.019	−0.025	**−0.057**	0.008	0.002	−0.026
π=0.2,ϕ=0.8	−0.013	−0.035	−0.045	−0.003	−0.035	−0.041	0.023	−0.003	−0.009
π=0.8,ϕ=0.2	0.006	−0.020	**−0.132**	0.015	−0.012	**−0.126**	0.045	0.017	**−0.095**
π=0.8,ϕ=0.8	**0.075**	−0.034	**−0.062**	**0.078**	−0.030	**−0.058**	**0.108**	−0.002	−0.029

*Note*: Model 1: No pre‐exposure period included in the model; Model 2: With pre‐exposure period of duration xϕ; Model 3: With pre‐exposure period of duration x. Median biases of absolute value at least 0.05 are in bold.

^a^ Monte Carlo standard error: 0.023.

^b^ Monte Carlo standard error: 0.010.

^c^ Monte Carlo standard error: 0.007.

**TABLE 2:** Median bias of $\hat{\beta }$ with risk period d=50, for contrasting values of the relative incidence exp(β), deferment interval duration x, and delay probabilities π and ϕ.

	exp(β)=0.5 ^a^			exp(β)=1 ^b^			exp(β)=2 ^c^		
	Model 1	Model 2	Model 3	Model 1	Model 2	Model 3	Model 1	Model 2	Model 3
x=15									
π=0.2,ϕ=0.2	0.005	0.004	−0.001	−0.003	−0.003	−0.008	0.007	0.006	0.003
π=0.2,ϕ=0.8	0.008	0.004	0.003	−0.001	−0.005	−0.006	0.011	0.007	0.005
π=0.8,ϕ=0.2	0.007	0.002	−0.014	0.000	−0.005	−0.023	0.011	0.006	−0.012
π=0.8,ϕ=0.8	0.022	0.003	−0.001	0.012	−0.005	−0.011	0.023	0.006	0.002
x=50									
π=0.2,ϕ=0.2	0.009	0.005	−0.009	0.000	−0.004	−0.017	0.011	0.008	−0.010
π=0.2,ϕ=0.8	0.018	0.003	−0.000	0.010	−0.003	−0.009	0.022	0.002	−0.001
π=0.8,ϕ=0.2	0.024	0.008	**−0.050**	0.017	0.001	**−0.062**	0.025	0.011	−0.048
π=0.8,ϕ=0.8	**0.065**	0.006	−0.008	**0.055**	−0.005	−0.021	**0.061**	0.005	−0.011
x=100									
π=0.2,ϕ=0.2	0.014	0.008	−0.019	0.007	0.000	−0.032	0.018	0.010	−0.022
π=0.2,ϕ=0.8	0.031	0.007	−0.002	0.024	−0.004	−0.013	0.034	0.004	−0.002
π=0.8,ϕ=0.2	**0.052**	0.024	**−0.096**	0.045	0.014	**−0.106**	**0.054**	0.024	**−0.096**
π=0.8,ϕ=0.8	**0.123**	0.009	−0.019	**0.114**	−0.001	−0.033	**0.121**	0.005	−0.022

*Note*: Model 1: No pre‐exposure period included in the model; Model 2: With pre‐exposure period of duration xϕ; Model 3: With pre‐exposure period of duration x. Median biases of absolute value at least 0.05 are in bold.

^a^ Monte Carlo standard error: 0.007.

^b^ Monte Carlo standard error: 0.005.

^c^ Monte Carlo standard error: 0.004.

**TABLE 3:** Median bias of $\hat{\beta }$ with risk period d=100, for contrasting values of the relative incidence exp(β), deferment interval duration x, and delay probabilities π and ϕ.

	exp(β)=0.5 ^a^			exp(β)=1 ^b^			exp(β)=2 ^c^		
	Model 1	Model 2	Model 3	Model 1	Model 2	Model 3	Model 1	Model 2	Model 3
x=15									
π=0.2,ϕ=0.2	0.000	−0.002	−0.008	−0.001	−0.002	−0.009	−0.002	−0.002	−0.007
π=0.2,ϕ=0.8	0.004	−0.003	−0.005	0.003	−0.003	−0.004	0.001	−0.002	−0.004
π=0.8,ϕ=0.2	0.003	−0.001	−0.021	0.003	−0.002	−0.002	0.002	−0.002	−0.021
π=0.8,ϕ=0.8	0.016	−0.002	−0.007	0.016	−0.004	−0.009	0.017	−0.002	−0.007
x=50									
π=0.2,ϕ=0.2	0.003	−0.003	−0.018	0.003	−0.003	−0.016	0.003	0.000	−0.015
π=0.2,ϕ=0.8	0.015	−0.001	−0.005	0.012	−0.001	−0.007	0.013	−0.002	−0.002
π=0.8,ϕ=0.2	0.021	0.004	**−0.063**	0.019	0.003	**−0.062**	0.020	0.003	**−0.062**
π=0.8,ϕ=0.8	**0.063**	−0.001	−0.015	**0.063**	−0.002	−0.018	**0.062**	0.000	−0.017
x=100									
π=0.2,ϕ=0.2	0.011	0.004	−0.027	0.009	0.002	−0.034	0.011	0.003	−0.031
π=0.2,ϕ=0.8	0.030	−0.000	−0.006	0.027	−0.003	−0.012	0.029	−0.004	−0.010
π=0.8,ϕ=0.2	**0.051**	0.018	**−0.114**	**0.051**	0.020	**−0.114**	**0.054**	0.023	**−0.110**
π=0.8,ϕ=0.8	**0.130**	0.003	−0.031	**0.127**	−0.001	−0.032	**0.127**	0.000	−0.033

*Note*: Model 1: No pre‐exposure period included in the model; Model 2: With pre‐exposure period of duration xϕ; Model 3: With pre‐exposure period of duration x. Median biases of absolute value at least 0.05 are in bold.

^a^ Monte Carlo standard error: 0.005.

^b^ Monte Carlo standard error: 0.004.

^c^ Monte Carlo standard error: 0.003.

The simulations confirm the theory: biases tend to be positive when no pre‐exposure interval is fitted, and negative when a pre‐exposure interval of duration x is included. Including a pre‐exposure interval of optimal length xϕ tends to reduce the bias. In addition, biases are small (absolute value below 0.050) when the deferment interval is short (x=15 days), and only become substantial (absolute value in excess of 0.100) with very long deferment intervals (x=100 days).

## 6 Recommendations

In practical applications, the duration x of the deferment period can be assessed from a histogram of the intervals between exposure and event. However, it is not usually possible to estimate ϕ, the proportion of long delays: typically, at most only informed guesses can be made. However, the theory developed above provides a basis for the following recommendations. In what follows, an exposure delay is deemed to be short if the risk period is included within the observation period; it is deemed to be long if the risk period is delayed beyond the end of the observation period. All exposure delays are caused by events occurring in a period x prior to the planned exposure.

- When exposure delays are short, then the standard SCCS model should be used, without including a pre‐exposure window.
- If there are long exposure delays, relative incidences will be biased upwards in the SCCS model with no pre‐exposure period. Thus, if the exposure is found not to be positively associated with the event, this finding is robust to the presence of long exposure delays. This may be verified using a sensitivity analysis (this is described below).
- If, on the other hand, the exposure is found to be positively associated with the event in the SCCS model with no pre‐exposure period, a sensitivity analysis can be undertaken to check whether this association could be the result of bias from long exposure delays.
- The sensitivity analyses referred to above involve fitting an SCCS model with pre‐exposure ‘risk’ period of duration x; this corresponds to the worst‐case scenario ϕ=1. Note that using this pre‐exposure period may overcorrect any bias from long exposure delays.
- When the deferment period x is substantial compared to the observation period, or when there are many long exposure delays, or when sensitivity analyses indicates that an association might be the result of bias from long exposure delays (in the sense that the model without a pre‐exposure window and the model with one of length x lead to conflicting conclusions), the SCCS model for event‐dependent exposures should be used in preference to the standard SCCS model.

## 7 Applications

In this section we illustrate how these recommendations might be implemented, with three examples. Further details of the three data sets may be found in Reference [1]; the original published data from which the examples are drawn have been jittered to preserve confidentiality. The SCCS models were fitted using the R package SCCS [11].

### 7.1 MMR Vaccine and Convulsion

**FIGURE 5:**
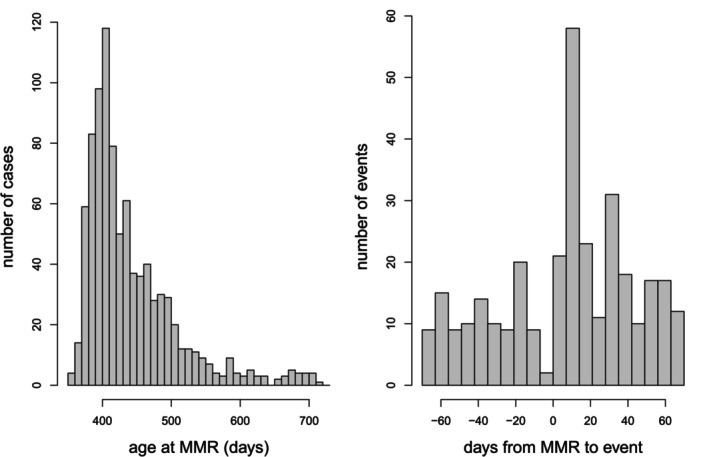
Left: Histogram of age at MMR vaccination. Right: Histogram of time interval from MMR vaccination to convulsion in the range −70 to +70 days (bin width: 7 days).

This example from the UK relates to convulsions and measles, mumps and rubella (MMR) vaccine given in the second year of life. The data comprise 988 convulsions, including 894 first convulsions and 94 recurrences. Every case received MMR. Figure 5 shows histograms of ages at vaccination and intervals from MMR vaccination to convulsion in the neighborhood of 0.

The histogram of intervals from MMR to convulsion shows a dip in numbers of convulsions in the 7 days prior to MMR. This suggests that MMR may be deferred in the week following a convulsion. Most delays are likely to be short: convulsions are not contraindications for vaccinations, and most children are likely to recover quickly. The histogram of ages at MMR has mode at about 13 months (about 400 days of age), in line with the recommended age for MMR vaccination in the UK; short delays are likely to have been accommodated within the long upper tail of the distribution. It is unlikely that many children will have vaccination deferred beyond the end of observation at 730 days. Thus, the primary model was the standard SCCS model with no pre‐exposure period, but we also did a sensitivity analysis by fitting SCCS models with pre‐exposure periods of 7 and, for good measure, 14 days. The risk period is 0 to 21 days, segmented in 3 weekly intervals: 0 to 7, 8 to 14, and 15 to 21 days. In all models, age effects were adjusted in 2‐month periods. The results are in Table 4.

**TABLE 4:** Relative incidence (95% confidence interval) of convulsion in post‐MMR risk periods, for SCCS models with contrasting pre‐exposure periods.

Pre‐exposure			
Period duration	None	7 Days	14 Days
Pre‐exposure	—	0.055 (0.008, 0.395)	0.364 (0.209, 0.633)
0–7 days	1.049 (0.676, 1.628)	1.002 (0.645, 1.555)	0.986 (0.635, 1.531)
8–14 days	3.252 (2.469, 4.282)	3.112 (2.362, 4.099)	3.065 (2.325, 4.040)
15–21 days	1.205 (0.785, 1.848)	1.157 (0.754, 1.775)	1.141 (0.743, 1.752)

The model without a pre‐exposure period indicates a three‐fold increased risk of convulsion in the second week after MMR, and no significant effect in the other two risk periods. The models with pre‐exposure risk periods of 7 or 14 days do not produce markedly different results. These models correspond to the extreme, and unlikely, situation in which all delays extend beyond 730 days (so x=7 or 14 and ϕ=1). We conclude that inferences based on the standard model with no pre‐exposure risk period are robust.

### 7.2 OPV and Intussusception

This example from Cuba relates to intussusception (telescoping of the bowel) and oral polio vaccine (OPV) [8]. The data comprise 273 intussusceptions in infants (aged 1 to 365 days) with at least one dose of OPV. OPV was administered as a 2‐dose schedule in vaccination campaigns that took place between January and June. Of the 273 cases, 256 received their first OPV dose and 224 received their second dose within the first year of life.

**FIGURE 6:**
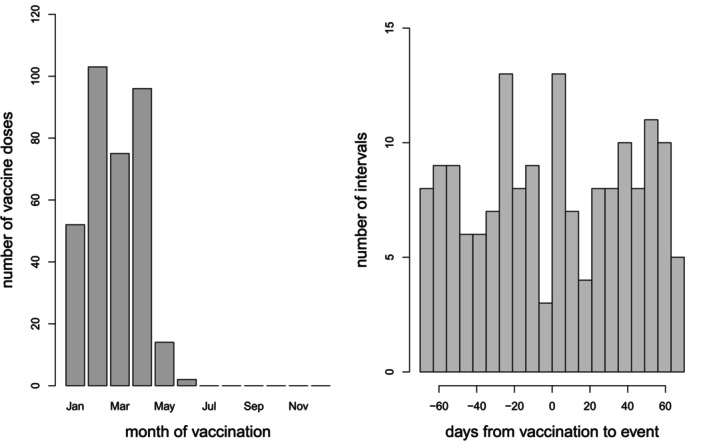
Left: Barchart of month of OPV. Right: Histogram of time interval from OPV to intussusception in the range −70 to +70 days (bin width: 7 days). Both plots show both doses combined.

A complication with vaccines involving more than one dose is how to handle overlaps between risk periods, this problem being compounded by the presence of pre‐exposure intervals. (When risk periods overlap, the SCCS model allocates the event to the period relating to the most recent dose.) The post‐OPV risk period of interest is 42 days, segmented as 0–7, 8–14, and 15–42 days. To avoid overlapping periods, we initially restricted the data by excluding infants who received both doses within the first year separated by less than 50 days: this restricted subgroup includes 204 cases. Within this group, the temporal distribution of OPV doses is shown in Figure 6. Also shown is the histogram of intervals from OPV (either dose) to intussusception in the neighborhood of 0.

The dip in the histogram in Figure 6 corresponding to the week before OPV suggests that some vaccinations are deferred in the 7 days after an intussusception. Intussusception is not a contra‐indication for OPV, and OPV coverage in Cuba at the time of the study was very high (97%), so polio vaccination is unlikely to have been routinely canceled after an event. However, especially owing to the seasonality of vaccination, it is possible that some doses were delayed beyond infancy. We therefore fitted two SCCS models to the restricted set of 204: one without a pre‐exposure period, and one with a 7‐day pre‐exposure period. We also fitted the SCCS model with no pre‐exposure period to the full dataset of 273 infants. All models included an age effect and a seasonal effect (both in monthly intervals). To simplify the presentation, we assumed a common effect at the two OPV doses (as it turns out, there is no significant difference between the doses). The results are in Table 5.

**TABLE 5:** Relative incidence (95% confidence interval) of intussusception in post‐OPV risk periods, for contrasting SCCS models.

Data used	Restricted	Restricted	Full
Pre‐expo period	None	7 Days	None
Pre‐exposure	—	0.287 (0.087, 0.943)	—
0–7 days	1.165 (0.669, 2.028)	0.941 (0.527, 1.677)	1.149 (0.713, 1.852)
8–14 days	0.788 (0.402, 1.544)	0.651 (0.321, 1.278)	0.974 (0.574, 1.653)
15–42 days	1.032 (0.571, 1.863)	0.848 (0.462, 1.559)	1.112 (0.679, 1.821)

*Note*: The restricted data include the 204 cases with OPV doses separated by at least 50 days; the full data include all 273 cases.

The SCCS model does not indicate a positive association between OPV and intussusception. Since long delays in exposure will tend to inflate the relative incidence, a sensitivity analysis is not strictly necessary to rule out a positive association. Nevertheless, including a 7‐day pre‐exposure period in the SCCS model confirms the direction of bias, should one exist. The analysis based on the entire data, rather than the restricted data, confirms the finding that there is no positive association.

### 7.3 Influenza Vaccine and GBS

**FIGURE 7:**
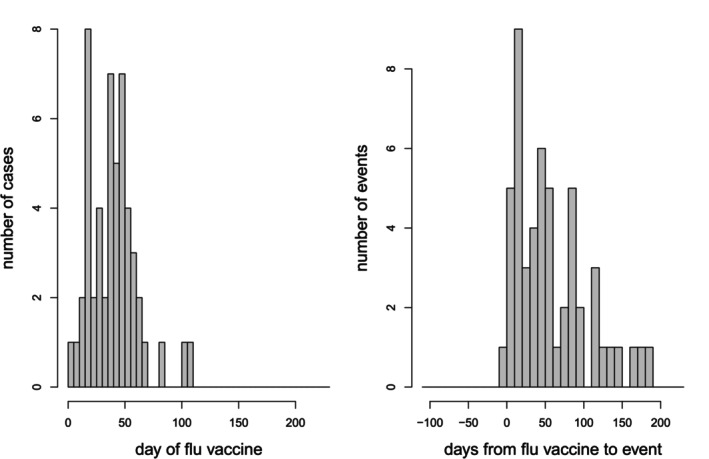
Left: Histogram of time of influenza vaccination. Right: Histogram of time interval from influenza vaccine to GBS.

The final example, from Italy, is one in which the methods described here may not apply. It relates to vaccination against influenza and Guillain Barré syndrome (GBS), a form of muscle weakness which may lead to paralysis [12]. The study includes 174 GBS cases arising during the 2010–11 influenza season, which stretched from 1 October 2010 to 15 May 2011; this time interval defines the observation period; 52 of these cases received influenza vaccine during that time, mainly during the first 10 weeks (70 days) as shown in Figure 7.

As shown in the histogram of time intervals from vaccination to GBS in Figure 7, only one GBS case was subsequently vaccinated (5 days after GBS onset). This is not due to there not having been any GBS cases early on in the observation period: there were 20 GBS cases in October 2010, all unvaccinated. This strongly suggests that influenza vaccination may have been discouraged (at least for the 2010–11 season) in persons having experienced GBS. Thus, delays in getting the vaccine are not restricted to a short deferment period after the event, which is the setting studied in the present paper, but apply to the entire post‐event period. This calls for a different SCCS model, developed specially for this setting [4].

However, because influenza vaccine is administered as a single dose, we could fit a standard SCCS model with a long pre‐exposure period, spanning all pre‐vaccination times. The last influenza vaccine was at 107 days, so the deferment period duration x=107 days will cover all pre‐vaccine periods. Also, since all delays are indefinite, ϕ=1; thus we used a pre‐exposure interval xϕ=107 days. The results of this model, together with those of the SCCS model with no pre‐exposure period, and the special SCCS model for event‐dependent exposures, are in Table 6. In all three models, the [0,42] day post‐vaccination risk period was segmented into 3 intervals: 0–7, 8–14, and 15–42 days; the models also included a temporal adjustment in months with a final 6‐week period.

**TABLE 6:** Relative incidence (95% confidence interval) of GBS in post‐influenza vaccination risk periods, for two standard SCCS models and the SCCS model for event‐dependent exposures.

SCCS model	Standard	Standard	Event‐dependent
Pre‐expo period	None	107 Days	Not applicable
Pre‐exposure	—	0.098 (0.013, 0.763)	—
0–7 days	1.444 (0.337, 6.181)	0.974 (0.223, 4.256)	1.058 (0.246, 4.561)
8–14 days	7.974 (3.740, 17.00)	5.501 (2.493, 12.14)	5.818 (2.680, 12.63)
15–42 days	1.644 (0.753, 3.587)	1.205 (0.541, 2.684)	1.217 (0.549, 2.699)

The estimates from the standard SCCS model with no pre‐exposure period are appreciably biased upwards. The standard SCCS model with 107‐day pre‐exposure period and the event‐dependent SCCS model give similar results in this case. Our preferred model is the event‐dependent SCCS model. It may be concluded that there was a strong positive association between the influenza vaccine used in the 2010–11 influenza season and GBS, concentrated within the second week after vaccination.

## 8 Discussion

We have sought to clarify the impact on the SCCS method of exposure delays initiated in a brief deferment period after the occurrence of an event. We have shown that such delays have little or no impact on the estimates of relative incidence associated with the exposure provided that the delayed risk periods still arise within the observation period. However, if the delays are longer, and move the risk period outside the observation period (or result in cancellation of the exposure), then the relative incidence will be biased upwards. We also clarified the impact of including a short pre‐exposure period, of duration equal to the deferment period, in the SCCS model. If delayed risk periods are still within the observation period, this will tend to introduce a negative bias. If, on the other hand, all delays move risk periods outside the observation period, an SCCS model including the deferment interval as a pre‐exposure period will correct the bias. Finally, we obtained a simple expression for the optimal pre‐exposure risk window duration: this is xϕ where x is the duration of the deferment interval and ϕ is the proportion of delayed exposures beyond the end of observation, given that a delay has occurred. These theoretical results were obtained in an idealized scenario; simulations support them in more realistic settings.

In practice, while x may be estimated from the pre‐exposure dip in a histogram of intervals between exposure and event, ϕ is not usually known. We therefore made a number of recommendations for undertaking sensitivity analyses (under the worst case scenario ϕ=1). In practice, any bias from short‐term deferment of exposure is likely to be small. This need not be the case if, on the other hand, the deferment period x is long, in which case the SCCS model for event‐dependent exposures should be used. These recommendations were illustrated by three contrasting case studies, which suggest that pre‐exposure periods should be used sparingly and preferably within sensitivity analyses.

Delayed vaccination following the adverse event of interest is related to but distinct from the well‐vaccinee effect. The difference is that, in the latter case, delayed exposures are due to the occurrence of prodromal symptoms for the event of interest, rather than the event itself. While the SCCS model controls for person‐specific confounders including individual frailty, it does not control for time‐varying confounding involving delayed vaccination. In the case of the well‐vaccinee effect, this typically generates a downward bias in the relative incidence [13]. The bias described in the present paper, on the other hand, may generate an upward bias for the SCCS model when some vaccinations are delayed beyond the end of observation. The advent of comprehensive recording of health information offers one possible avenue of further development. If data on vaccination appointments as well as vaccination dates were available for cases, it would be possible to study directly the event, vaccination appointment, and vaccination delivery processes and their interaction, and to estimate the parameters x, π, and ϕ directly from data. It would also be possible to undertake intention‐to‐treat SCCS analyses based on date of vaccination appointment rather than date of actual vaccination. These possibilities suggest some avenues for further research.

## Funding

The authors have nothing to report.

## Conflicts of Interest

The authors declare no conflicts of interest.

## Supporting information


**Data S1:** sim70566‐sup‐0001‐Supinfo.pdf.

## Data Availability

The data we used for the three examples are available within R package SCCS under filenames febdat, intdat, and gbsdat, in The Comprehensive R Archive Network (CRAN) at DOI: 10.32614/CRAN.package.SCCS.
