# Supplementary material for: Delayed Exposures and Pre‐Exposure Periods in Self‐Controlled Case Series Studies
Source: Stat Med. 2026 Apr 27;45:e70566. doi: 10.1002/sim.70566 (PMC13120864; doi:10.1002/sim.70566)
Supplement: Supplementary file 1 — Data S1: sim70566‐sup‐0001‐Supinfo.pdf. [file SIM-45-0-s001.pdf]

Supplementary Material
Delayed exposures and pre-exposure periods in self-controlled case series studies

Heather Whitaker, Yonas Ghebremichael Weldeselassie, Paddy Farrington

March 4, 2026

1 Motivating scenarios: Delayed exposure risk windows partially observed

We consider here a third scenario in which events occurring within x days before planned exposure result in the postponement of the exposure for y days, where y could be short or long, but the delayed exposures result in risk windows that partially fall beyond the end of the observation period, as illustrated in Figure 1 below.

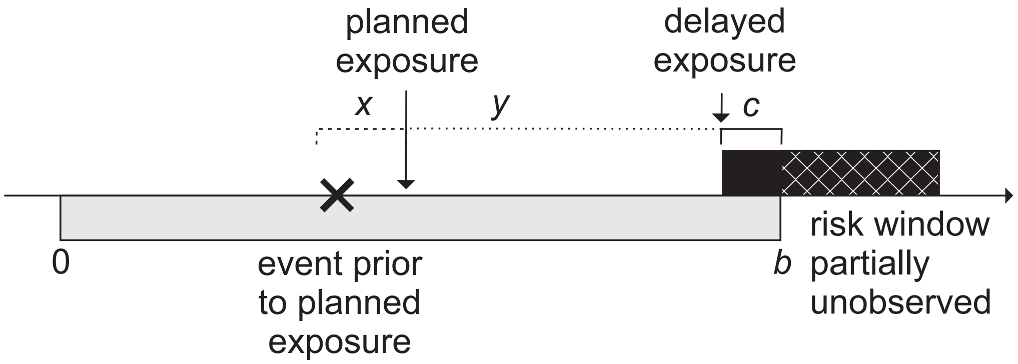

Figure 1: Delay by length of time y such that all delayed exposure risk windows fall partially within the observation period.

For simplicity, we assume that all cases start with exactly the same observation period of length b, and have a planned risk period of length d. As before, N_0 is the total number of cases whose event fell in the baseline period (including those with delayed exposure), but delay such that the risk period is truncated or partially observed occurs in N_0^{del} cases ($N_0^{del} \leq N_0$). The SCCS likelihood

is then

$$L = N_1 \left(\frac{de^{\beta}}{de^{\beta} + b - d} \right) \times (N_0 - N_0^{del}) \left(\frac{b - d}{de^{\beta} + b - d} \right) \times N_0^{del} \left(\frac{b - c}{ce^{\beta} + b - c} \right),$$

where c is the length of the truncated risk window in cases with delayed exposure, as illustrated in Figure 1. The corresponding maximum likelihood estimate for the relative incidence is

$$\begin{aligned} e^{\hat{\beta}} = \frac{1}{2cdN_0} \Big\{ & bcN_1 - bcN_0^{del} - bdN_0 + bdN_0^{del} + cdN_0 - cdN_1 \\ & + \sqrt{4cdN_0 N_1 (b^2 - bc - bd + cd) + (bcN_1 - bcN_0^{del} - bdN_0 + bdN_0^{del} + cdN_0 - cdN_1)^2} \Big\}. \end{aligned} \quad (1)$$

If a pre-exposure window of length p is added, b is replaced by $b - p$ in the above.

In practice, such a scenario would occur frequently when exposure risk windows are of indefinite length or are long in relation to the length of the observation period. The length of a truncated risk window c would differ between individuals, and d would also differ between individuals when risk windows are of indefinite length. Models for delayed exposures where risk windows are truncated in this way become complicated, and are not considered further in this paper.

2 Simulations: Inclusion of age effects

We present the simulation results obtained with increasing age effects (Supplementary Tables 1 to 3) and decreasing age effects (Supplementary Tables 4 to 6), as described in the main paper. The Monte Carlo standard errors were the same as those obtained with no age effects (see main paper).

Table 1: Increasing age effect: Median bias of $\hat{\beta}$ with risk period $d = 15$, for contrasting values of the relative incidence $\exp(\beta)$, deferment interval duration x, and delay probabilities π and ϕ. Model 1: no pre-exposure period included in the model; model 2: with pre-exposure period of duration $x\phi$; model 3: with pre-exposure period of duration x. Median biases of absolute value at least 0.05 are in bold.

	$\exp(\beta) = 1$			$\exp(\beta) = 2$		
	Model 1	Model 2	Model 3	Model 1	Model 2	Model 3
$x = 15$						
$\pi = 0.2, \phi = 0.2$	-0.029	-0.031	-0.035	-0.008	-0.009	-0.013
$\pi = 0.2, \phi = 0.8$	-0.025	-0.032	-0.032	-0.004	-0.009	-0.010
$\pi = 0.8, \phi = 0.2$	-0.025	-0.030	-0.046	-0.003	-0.007	-0.024
$\pi = 0.8, \phi = 0.8$	-0.012	-0.030	-0.034	0.010	-0.008	-0.013
$x = 50$						
$\pi = 0.2, \phi = 0.2$	-0.025	-0.029	-0.041	-0.004	-0.008	-0.023
$\pi = 0.2, \phi = 0.8$	-0.015	-0.028	-0.031	0.006	-0.005	-0.010
$\pi = 0.8, \phi = 0.2$	-0.010	-0.024	**-0.082**	0.011	-0.005	**-0.062**
$\pi = 0.8, \phi = 0.8$	0.029	-0.028	-0.042	0.049	-0.006	-0.022
$x = 100$						
$\pi = 0.2, \phi = 0.2$	-0.018	-0.023	-0.051	0.005	-0.001	-0.031
$\pi = 0.2, \phi = 0.8$	-0.001	-0.024	-0.029	0.019	-0.005	-0.016
$\pi = 0.8, \phi = 0.2$	0.020	-0.009	**-0.127**	0.039	0.012	**-0.107**
$\pi = 0.8, \phi = 0.8$	**0.089**	-0.023	**-0.052**	**0.109**	-0.006	-0.032

Table 2: Increasing age effect: Median bias of $\hat{\beta}$ with risk period $d = 50$, for contrasting values of the relative incidence $\exp(\beta)$, deferment interval duration x, and delay probabilities π and ϕ. Model 1: no pre-exposure period included in the model; model 2: with pre-exposure period of duration $x\phi$; model 3: with pre-exposure period of duration x. Median biases of absolute value at least 0.05 are in bold.

	$\exp(\beta) = 1$			$\exp(\beta) = 2$		
	Model 1	Model 2	Model 3	Model 1	Model 2	Model 3
$x = 15$						
$\pi = 0.2, \phi = 0.2$	0.001	-0.001	-0.004	-0.000	-0.001	-0.006
$\pi = 0.2, \phi = 0.8$	0.003	-0.003	-0.003	0.003	-0.001	-0.003
$\pi = 0.8, \phi = 0.2$	0.004	-0.001	-0.021	0.004	-0.001	-0.018
$\pi = 0.8, \phi = 0.8$	0.018	0.001	-0.005	0.016	-0.002	-0.006
$x = 50$						
$\pi = 0.2, \phi = 0.2$	0.003	-0.000	-0.015	0.004	0.000	-0.015
$\pi = 0.2, \phi = 0.8$	0.014	-0.002	-0.005	0.014	0.000	-0.004
$\pi = 0.8, \phi = 0.2$	0.018	0.004	**-0.059**	0.020	0.005	**-0.056**
$\pi = 0.8, \phi = 0.8$	**0.059**	0.000	-0.016	**0.060**	0.001	-0.014
$x = 100$						
$\pi = 0.2, \phi = 0.2$	0.011	0.004	-0.030	0.014	0.005	-0.028
$\pi = 0.2, \phi = 0.8$	0.028	-0.001	-0.011	0.028	-0.001	-0.007
$\pi = 0.8, \phi = 0.2$	0.049	0.018	**-0.107**	**0.050**	0.018	**-0.104**
$\pi = 0.8, \phi = 0.8$	**0.121**	0.001	-0.030	**0.121**	0.003	-0.029

Table 3: Increasing age effect: Median bias of $\hat{\beta}$ with risk period $d = 100$, for contrasting values of the relative incidence $\exp(\beta)$, deferment interval duration x, and delay probabilities π and ϕ. Model 1: no pre-exposure period included in the model; model 2: with pre-exposure period of duration $x\phi$; model 3: with pre-exposure period of duration x. Median biases of absolute value at least 0.05 are in bold.

	$\exp(\beta) = 1$			$\exp(\beta) = 2$		
	Model 1	Model 2	Model 3	Model 1	Model 2	Model 3
$x = 15$						
$\pi = 0.2, \phi = 0.2$	-0.006	-0.008	-0.013	-0.005	-0.007	-0.010
$\pi = 0.2, \phi = 0.8$	-0.002	-0.010	-0.010	-0.002	-0.006	-0.007
$\pi = 0.8, \phi = 0.2$	-0.002	-0.007	-0.027	0.000	-0.005	-0.025
$\pi = 0.8, \phi = 0.8$	0.011	-0.008	-0.013	0.013	-0.006	-0.010
$x = 50$						
$\pi = 0.2, \phi = 0.2$	-0.001	-0.006	-0.020	-0.001	-0.004	-0.023
$\pi = 0.2, \phi = 0.8$	0.007	-0.005	-0.009	0.010	-0.006	-0.010
$\pi = 0.8, \phi = 0.2$	0.015	-0.001	**-0.067**	0.017	0.000	**-0.065**
$\pi = 0.8, \phi = 0.8$	**0.061**	-0.003	-0.019	**0.058**	-0.006	-0.022
$x = 100$						
$\pi = 0.2, \phi = 0.2$	0.007	-0.001	-0.036	0.005	-0.001	-0.035
$\pi = 0.2, \phi = 0.8$	0.023	-0.006	-0.015	0.025	-0.003	-0.012
$\pi = 0.8, \phi = 0.2$	**0.052**	0.019	**-0.117**	**0.051**	0.018	**-0.113**
$\pi = 0.8, \phi = 0.8$	**0.129**	-0.001	-0.034	**0.126**	-0.001	-0.034

Table 4: Decreasing age effect: Median bias of $\hat{\beta}$ with risk period $d = 15$, for contrasting values of the relative incidence $\exp(\beta)$, deferment interval duration x, and delay probabilities π and ϕ. Model 1: no pre-exposure period included in the model; model 2: with pre-exposure period of duration $x\phi$; model 3: with pre-exposure period of duration x. Median biases of absolute value at least 0.05 are in bold.

	$\exp(\beta) = 1$			$\exp(\beta) = 2$		
	Model 1	Model 2	Model 3	Model 1	Model 2	Model 3
$x = 15$						
$\pi = 0.2, \phi = 0.2$	-0.023	-0.024	-0.028	-0.022	-0.023	-0.025
$\pi = 0.2, \phi = 0.8$	-0.019	-0.023	-0.025	-0.017	-0.020	-0.023
$\pi = 0.8, \phi = 0.2$	-0.018	-0.024	-0.041	-0.017	-0.022	-0.037
$\pi = 0.8, \phi = 0.8$	-0.008	-0.024	-0.028	-0.003	-0.021	-0.025
$x = 50$						
$\pi = 0.2, \phi = 0.2$	-0.019	-0.022	-0.035	-0.017	-0.020	-0.034
$\pi = 0.2, \phi = 0.8$	-0.010	-0.022	-0.025	-0.006	-0.017	-0.022
$\pi = 0.8, \phi = 0.2$	-0.004	-0.018	**-0.077**	-0.004	-0.018	**-0.073**
$\pi = 0.8, \phi = 0.8$	0.037	-0.022	-0.036	0.036	-0.022	-0.035
$x = 100$						
$\pi = 0.2, \phi = 0.2$	-0.013	-0.020	-0.047	-0.007	-0.013	-0.044
$\pi = 0.2, \phi = 0.8$	0.005	-0.017	-0.027	0.007	-0.016	-0.024
$\pi = 0.8, \phi = 0.2$	0.025	-0.002	**-0.122**	0.028	0.002	**-0.117**
$\pi = 0.8, \phi = 0.8$	**0.094**	-0.020	-0.046	**0.096**	-0.017	-0.045

Table 5: Decreasing age effect: Median bias of $\hat{\beta}$ with risk period $d = 50$, for contrasting values of the relative incidence $\exp(\beta)$, deferment interval duration x, and delay probabilities π and ϕ. Model 1: no pre-exposure period included in the model; model 2: with pre-exposure period of duration $x\phi$; model 3: with pre-exposure period of duration x. Median biases of absolute value at least 0.05 are in bold.

	$\exp(\beta) = 1$			$\exp(\beta) = 2$		
	Model 1	Model 2	Model 3	Model 1	Model 2	Model 3
$x = 15$						
$\pi = 0.2, \phi = 0.2$	-0.008	-0.010	-0.012	0.000	-0.002	-0.006
$\pi = 0.2, \phi = 0.8$	-0.004	-0.009	-0.008	0.003	-0.002	-0.003
$\pi = 0.8, \phi = 0.2$	-0.003	-0.008	-0.027	0.004	-0.001	-0.020
$\pi = 0.8, \phi = 0.8$	0.010	-0.009	-0.012	0.017	-0.003	-0.007
$x = 50$						
$\pi = 0.2, \phi = 0.2$	-0.004	-0.006	-0.022	0.003	-0.002	-0.017
$\pi = 0.2, \phi = 0.8$	0.006	-0.007	-0.011	0.012	-0.002	-0.007
$\pi = 0.8, \phi = 0.2$	0.013	-0.000	**-0.064**	0.020	0.002	**-0.060**
$\pi = 0.8, \phi = 0.8$	**0.052**	-0.009	-0.025	**0.061**	-0.004	-0.020
$x = 100$						
$\pi = 0.2, \phi = 0.2$	0.003	-0.001	-0.031	0.012	0.003	-0.028
$\pi = 0.2, \phi = 0.8$	0.020	-0.003	-0.010	0.027	-0.004	-0.009
$\pi = 0.8, \phi = 0.2$	0.045	0.016	**-0.114**	**0.053**	0.020	**-0.109**
$\pi = 0.8, \phi = 0.8$	**0.117**	-0.002	-0.033	**0.123**	-0.000	-0.031

Table 6: Decreasing age effect: Median bias of $\hat{\beta}$ with risk period $d = 100$, for contrasting values of the relative incidence $\exp(\beta)$, deferment interval duration x, and delay probabilities π and ϕ. Model 1: no pre-exposure period included in the model; model 2: with pre-exposure period of duration $x\phi$; model 3: with pre-exposure period of duration x. Median biases of absolute value at least 0.05 are in bold.

	$\exp(\beta) = 1$			$\exp(\beta) = 2$		
	Model 1	Model 2	Model 3	Model 1	Model 2	Model 3
$x = 15$						
$\pi = 0.2, \phi = 0.2$	-0.006	-0.008	-0.011	0.002	0.002	-0.004
$\pi = 0.2, \phi = 0.8$	-0.002	-0.004	-0.007	0.006	0.001	-0.001
$\pi = 0.8, \phi = 0.2$	-0.001	-0.006	-0.028	0.007	0.001	-0.019
$\pi = 0.8, \phi = 0.8$	0.014	-0.007	-0.013	0.022	0.000	-0.005
$x = 50$						
$\pi = 0.2, \phi = 0.2$	-0.002	-0.005	-0.022	0.007	0.001	-0.013
$\pi = 0.2, \phi = 0.8$	0.009	-0.004	-0.011	0.017	0.004	-0.001
$\pi = 0.8, \phi = 0.2$	0.016	0.000	**-0.071**	0.025	0.009	**-0.063**
$\pi = 0.8, \phi = 0.8$	**0.063**	-0.008	-0.026	**0.072**	0.003	-0.015
$x = 100$						
$\pi = 0.2, \phi = 0.2$	0.008	0.001	-0.037	0.016	0.007	-0.028
$\pi = 0.2, \phi = 0.8$	0.025	-0.002	-0.015	0.035	0.006	-0.005
$\pi = 0.8, \phi = 0.2$	**0.054**	0.019	**-0.126**	**0.063**	0.027	**-0.117**
$\pi = 0.8, \phi = 0.8$	**0.133**	-0.004	-0.040	**0.143**	0.008	-0.028
